# Dr. Nathan O. Harris

**Published:** 1896-04

**Authors:** 


### DR. NATHAN O. HARRIS.

### MEMORIAL ADOPTED AT A MEETING OF THE ATLANTA SOCIETY OF MEDICINE.

The following memorial on the death of Dr. N. O. Harris was adopted by the Atlanta Society of Medicine at a meeting held March 18th:

The life most resplendent with beauty and usefulness is the one which places self in the remote background and advances the pleasures and comforts of others far above anything pertaining to his own personal happiness. He who truly cherishes and graciously assists struggling humanity in its upward course; who listens to the plea of the sufferer, be it man, woman, or child, in station high or low, and comforts, by gift or affectionate advice, the hearts bruised and bleeding, merits recognition more remunerative than worldly hands can dispense.

These and many other bright traits were present in the character of our esteemed and lamented colleague, Dr. Nathan Overton Harris, who departed life Friday, March 6, 1896, aged forty four years. He was born in Culpepper county, Virginia, December 4, 1851, the son of James O. and Elizabeth Brown Harris. During the late war, he removed with his father to Atlanta, where he attained his education. In 1868, he attended school in Covington, Kentucky. He read medicine under Dr. Thomas S. Powell and graduated from the Southern Medical College in 1881. He then practiced medicine in Atlanta for eighteen months; during three months of this time he had charge of the smallpox hospital, treating 200 patients for this dread disease. In 1883 he graduated from Bellevue Hospital Medical College and returned to Atlanta to again prosecute his chosen work. In 1888 he was elected assistant to the chair of obstetrics and diseases of women and children in the Atlanta Medical College. He was a member in excellent standing in several medical organizations, viz: the Atlanta Society of Medicine, of which he served as president in 1886, and corresponding secretary for a number of years; Medical Association of Georgia; American Medical Association; National Association of Railway Surgeons; besides being medical examiner for a number of life insurance companies. He was also surgeon for the Georgia, Carolina and Northern Railroad division of the Seaboard Air line; surgeon for the Fourth battalion, Georgia Volunteers; physician for sixth ward of Atlanta; a consistent member of St. Phillip's Episcopal church. He was married April 8, 1885 to Lula S., daughter of Rufus S. Tucker, of Raleigh, North Carolina, who died April 23, 1886.

In the death of Dr. Harris, a true friend, genial gentleman, an honorable man, and a good physician has been taken from us, and a void created which must long remain vacant. It can truly be said of him that he had no enemies; all who spoke of him used the most affectionate language. He had the peculiar ability to make friends, and when once his friend there was no resistance to his personal magnetism, which drew one closer and closer to him as a more thorough knowledge of his inner nature developed. He was one of the few men whom men could love with genuine affection and expect a reciprocation of this feeling from such a benignant nature. His bright affable manner, his genial disposition, his useful professional advice will long be missed; but He who dispenses all things wisely has seen fit to remove him from us and we must submit to His divine will.

(Signed) C. C. GREENE, Chairman,
J. B. S. HOLMES,
H. P. COOPER,
W. S. ELKIN.

